# Factors associated with the inter-facility transfer of inpatients in Sichuan province, China

**DOI:** 10.1186/s12913-019-4153-7

**Published:** 2019-05-23

**Authors:** Linxin Liu, Chaojie Liu, Zhanqi Duan, Jingping Pan, Min Yang

**Affiliations:** 1 West China School of Public Health, Sichuan University, Chengdu, Sichuan People’s Republic of China; 2 School of Psychology and Public Health, La Trobe University, Melbourne, VIC 3086 Australia; 3 Health and Family Planning Information Centre of Sichuan Province, Chengdu, Sichuan People’s Republic of China; 4 West China School of Public Health, West China Research Center for Rural Health Development, Sichuan University, Chengdu, 610041 Sichuan People’s Republic of China; 5 Faculty of Health, Arts and Design, Swinburne University of Technology, Melbourne, Australia

**Keywords:** Inter-facility transfer, Inpatient care, Health policy, Health insurance, General hospital

## Abstract

**Background:**

The overuse of tertiary hospitals and underuse of primary care facilities has been one of the key reasons leading to fast health expenditure increase and health service utilization inequity in China. Recent health care reform in China tries to enforce a patient transfer system to make the health services utilization more efficient. This study examined the pattern and associated factors of inter-facility transfer of inpatients in Sichuan province of Western China.

**Methods:**

Patient discharge records (*n* = 1,490,695) from 604 general hospitals during the period of April to June 2015 in Sichuan were extracted from the front page of the medical records system with individual information on demographics, insurance coverage, diagnoses, hospitals admitted and discharge type. We calculated the percentage of inpatients transferring to other health facilities, the Inter-Facility Transfer Rate (IFTR) with adjustment for Charlson Comorbidity Index (CCI). Multi-level logistic regression models were established to identify factors associated with IFTRs.

**Results:**

A small number of tertiary hospitals (*n* = 75, 12.41%) shared 51.71% (*n* = 770,823) of all admitted cases while a large number of primary/unrated hospitals (*n* = 321, 53.15%) shared only 8.15%. The overall CCI-adjusted IFTR was 2.08% with 3.73% among secondary hospitals, 1.87% among tertiary hospitals and 1.30% among primary/unrated hospitals. Uninsured patients (OR = 1.13) and those with a lower level of insurance entitlements (OR = 1.12 for the New Rural Cooperative Medical Scheme and the Basic Medical Insurance for Urban Residents) were more likely to experience inter-facility transfer than those with a higher level of insurance entitlements (the Basic Medical Insurance for Urban Employees).

**Conclusion:**

The level of IFTR in general hospitals in Sichuan is low, which is associated with the level of hospitals and insurance entitlements. Further studies are needed to better understand how patients and health care providers respond to different insurance policies and make decisions on inter-facility transfer.

**Electronic supplementary material:**

The online version of this article (10.1186/s12913-019-4153-7) contains supplementary material, which is available to authorized users.

## Background

Inter-facility transfer refers to any patient transfer between two health care facilities for specific procedures, continuing medical care or rehabilitation. The bond between health care facilities in a regional medical network is strong in many developed countries such as Australia, the USA, and the UK. Patient safety in terms of mortality is a major concern in inter-facility transfer studies [1, 2]. Many practice guidelines have been developed in developed countries, requiring both the referring and receiving facilities to take actions to ensure the safety, continuity and coordination of patient care during the transfer process, which includes the initial decision of patient transfer [3].

In China, the health service delivery system comprises hospitals, primary care institutions and public health institutions [4]. The hospital sector is dominated by public hospitals [5]. The hospital accreditation system run by the government classifies hospitals into three levels: primary, secondary and tertiary, based on their size, scope and capacity of services. This system applies to both general and specialist hospitals. Tertiary hospitals usually have more than 500 beds, compared to 100–499 beds for secondary hospitals and 20–99 beds for primary hospitals [6]. Higher levels of hospitals are staffed by better-educated health workers, have more advanced medical equipment, and are able to deliver a wider range of services. The price schedule set up by the government is also linked to the level of hospitals: higher prices for higher levels of hospitals.

In China, patients are entitled to choose any local health facilities for services [7]. Despite a higher proportion of out-of-pocket payments, patients rely heavily on large hospitals, even for minor and non-urgent illness [8]. The overuse of tertiary hospitals and the underuse of primary and secondary facilities has been blamed for inflating health expenditure [8]. This problem has become one of the major targets of the recent health system reform.

In 2009, the Chinese government launched a new round of health reform, with an aim to develop a healthcare system with good accessibility, affordability, safety and quality. The reform envisaged a tiered health care delivery system (THCDS), which requires “first contact at primary care facilities and coordinated referral practices” [9]. Patients in urgent or critical conditions are supposed to be transferred from primary care facilities to higher levels of hospitals or from one tertiary hospital to another with strength in the relevant area of specialist care; whereas patients with chronic conditions and those in a stable condition are supposed to be managed by primary care facilities [10]. In 2015, the State Council issued guidelines for developing the THCDS [11]. However, no primary care gatekeeping mechanisms are included in the guidelines. A strong focus has been placed on structural adjustments of health care facilities, including strengthening primary care and functional specifications for different levels of health facilities [4].

Patient referral and transfer arrangements are shaped by factors associated with patients, health facilities and third-party payers such as medical insurance [12, 13]. Unfortunately, the THCDS proposal has not been well endorsed by patients. Studies show that patients are concerned about the low capacity and poor quality of care delivered by primary care facilities, preventing them from using primary care facilities as first contact or being transferred back from higher levels of hospitals [10]. Patients and their family always weigh the need for treatments against a range of considerations, such as the price of different treatment regimes, insurance reimbursements, and the prospect of a full recovery. Their decisions are likely to be shaped by their financial capacity, educational attainment, and cultural background.

Although some health facilities signed agreements for patient referral arrangements in response to the national policy, the implementation is challenging due to a lack of enforcement and punitive measures [14]. Over the past few decades, health facilities in China have been exposed to intense market competition. They have increasingly relied on revenues generated from fee for services. The government budget comprised less than 9% of hospital revenues over the last decade (Table 1). In this context, health facilities at lower levels might still refer/transfer patients to a higher level facility to address concerns for patient safety. Indeed, the service capacity of primary care facilities has been alarmingly low in China and the majority of doctors work in medium- and large-sized hospitals (Table 1). However, it is less likely for hospitals to refer/transfer patients to another hospital or a lower level facility because doing so simply means a loss of revenue.

**Table 1:** Number (percentage) of resources and services across three levels of hospitals in China (2002–2015)^a^

Resources/services		2002	2005	2008	2011	2014	2015
No. of hospitals (%)	Primary/unrated	2674 (30.22)	2714 (30.80)	4989 (38.49)	5636 (41.74)	7009 (44.32)	8759 (47.67)
	Secondary	5198 (58.74)	5156 (58.50)	6780 (52.31)	6468 (47.90)	6850 (43.32)	7494 (40.78)
	Tertiary	977 (11.04)	943 (10.70)	1192 (9.20)	1399 (10.36)	1954 (12.36)	2123 (11.55)
No. of registered doctors (%)	Primary/unrated	–	–	–	76,775 (7.12)	91,519 (6.88)	108,528 (7.49)
	Secondary	–	–	–	554,434 (51.39)	583,433 (43.83)	615,889 (42.50)
	Tertiary	–	–	–	447,758 (41.50)	656,112 (49.29)	724,782 (50.01)
No. of beds (%)	Primary/unrated	–	147,522 (8.52)	233,018 (9.26)	277,233 (8.63)	387,207 (8.96)	481,876 (10.20)
	Secondary	–	986,851 (57.00)	1,425,406 (56.66)	1,710,135 (53.26)	2,053,896 (47.55)	2,196,748 (46.48)
	Tertiary	–	597,051 (34.48)	857,304 (34.08)	1,223,584 (38.11)	1,878,267 (43.48)	2,047,819 (43.33)
No. of admitted patients (10,000) (%)	Primary/unrated	–	207.1 (5.28)	392.2 (5.78)	535.8 (5.46)	798 (5.66)	965.2 (6.47)
	Secondary	–	2297.7 (58.58)	4061.3 (59.90)	5567.4 (56.69)	7005.7 (49.70)	7121.2 (47.74)
	Tertiary	–	1417.6 (36.14)	2326.8 (34.32)	3717.3 (37.85)	6291 (44.63)	6828.9 (45.78)
Inpatient expense per capita in public hospitals (RMB) (%)	Primary/unrated	–	–	–	3121.3 (16.76)	3737.1 (17.84)	3844.5 (17.63)
	Secondary	–	–	–	4564.2 (24.51)	5114.6 (24.41)	5358.2 (24.58)
	Tertiary	–	–	–	10,935.9 (58.73)	12,100.2 (57.75)	12,599.3 (57.79)
Government budget as a percentage of revenue in public hospitals		7.35	5.09	6.6	7.76	6.99	8.19

^a^ Data source: Statistical reports from the National Health and Family Planning Commission of China

Anecdotal evidence shows that medical insurance policies also have a significant impact on patient referral/transfer arrangements [15]. It is a common practice in China that patients are charged a higher price and given a lower percentage of insurance reimbursement for services in tertiary hospitals compared with those in secondary and primary facilities. However, there is no evidence available to demonstrate whether and how such policies work in encouraging patients to change their preference. Tertiary hospital services have continued to grow at a speed higher than the secondary and primary care facilities (Table 1).

It is important to understand how hospitals refer/transfer patients before appropriate interventions can be developed. This study aimed to examine inter-facility transfer activities for hospital inpatients in Sichuan province, using a large database of patient records maintained by the provincial health authority.

## Methods

A cross-sectional study was undertaken. The research questions of this study were: (1) what percentage of patients admitted to hospitals experienced inter-facility transfer? (2) what were the factors associated with the inter-facility transfer of inpatients?

### Setting

The study was conducted in Sichuan province covering hospital separation data from April to June 2015. Sichuan is located in the southwest of China, with a population of over 81 million. It had a GDP per capita of 35,128 Yuan RMB in 2014, ranking No. 23 of all 31 provinces in China. A great disparity in economic development exists in Sichuan. The 21 prefectures in Sichuan are divided into three economic zones [16], with a GDP per capita ranging from 13,756 to 70,646 Yuan RMB in 2014 (Table 2).

**Table 2:** GDP in Chinese RMB and medical resources in 21 prefectures of Sichuan in 2014^a^

Economic zone	Prefectures	GDP (billion RMB)	GDP Per Capita (RMB)	Population (10,000)	No. of hospitals per km^2^	No. of hospitals per 1000 persons	No. of beds per 1000 persons
One	Panzhihua	87.085	70,646	123.20	0.0039	0.0235	7.7914
One	Chengdu	1005.659	70,019	1442.80	0.0412	0.0346	7.4876
Two	Deyang	151.565	43,091	351.10	0.0125	0.0211	5.4452
Two	Zigong	107.340	39,145	274.58	0.0155	0.0248	5.9713
Two	Leshan	120.759	37,125	325.00	0.0075	0.0292	5.8397
Two	Mianyang	157.989	33,558	473.90	0.0040	0.0171	6.4900
Two	Ziyang	119.560	33,592	354.72	0.0064	0.0144	5.8770
Two	Meishan	94.489	31,664	298.97	0.0071	0.0171	5.2948
Two	Yibin	144.381	32,318	447.00	0.0064	0.0190	5.6065
Two	Yaan	46.241	30,052	154.37	0.0027	0.0266	7.0040
Two	Neijiang	115.677	31,024	373.26	0.0119	0.0171	5.3113
Two	Luzhou	125.973	29,655	425.00	0.0084	0.0242	5.3332
Two	Guangan	91.961	28,489	323.16	0.0080	0.0158	4.0887
Two	Suining	80.955	24,691	328.25	0.0113	0.0183	4.8210
Two	Dazhou	134.783	24,411	553.05	0.0046	0.0137	4.0674
Two	Nanchong	143.202	22,639	633.38	0.0091	0.0180	4.6844
Two	Guangyuan	56.619	22,117	257.50	0.0032	0.0202	6.5060
Two	Bazhong	45.666	13,756	332.21	0.0041	0.0154	4.3972
Three	Ganzi	20.681	18,096	114.79	0.0003	0.0357	4.4107
Three	Aba	24.779	27,043	92.03	0.0004	0.0380	4.6420
Three	Liangshan	131.430	28,556	461.93	0.0017	0.0219	4.2797
	Sichuan	2853.670	35,128	8140.2	0.0037	0.0224	5.6459

^a^Data source: Statistical reports from Sichuan Health and Family Planning Commission and Sichuan Statistics Yearbook

The Sichuan government initiated the THCDS reform in September 2014 [17]. In the proposed THCDS, county hosptials, township/community health centers, and village clinics are categorized as primary care facilities, although the majority of county hospitals are actually secondary hospitals. Capacity building programs in relation to these facilities were developed with an intention to attract more patients to primary care facilities. In 2015, the Sichuan health authority introduced two indicators to monitor the progress of the THCDS: number of referred/transferred patients and patient referral/transfer rates [18].

Sichuan had 1942 hospitals by the end of 2015, including 1291 general hospitals and 651 non-general hospitals (e.g. specialist hospitals and hospitals of Chinese medicine). This study extracted data from 863 general hospitals in the province from April to June 2015, which covered 330 public hospitals and 533 private hospitals. The extracted data contained 1.57 million patient discharge records (61.57% of all cases over the same period). Non-general hospitals were excluded from the study.

### Data source

Data were extracted from the front page of inpatient medical records, which included socio-demographic characteristics (age, sex, ethnicity) and the insurance coverage of the patient, the location, ownership and accredited level of the treating hospitals, services related to the episode of care (diagnoses, surgery), and discharge destinations.

**Fig. 1:**
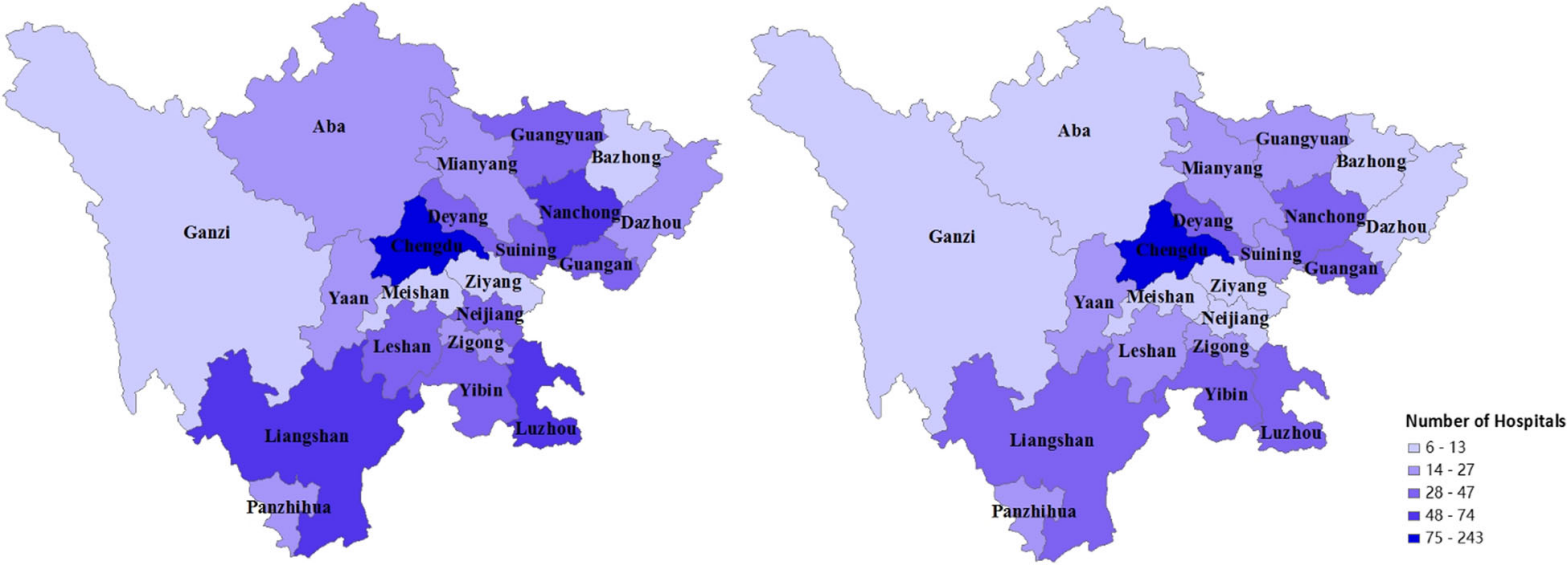
Geographic distribution of hospitals across the 21 prefectures in Sichuan province. Notes: Map on the left shows the distribution of all hospitals in the database and on the right the distribution of the participating hospitals

The item “discharge destination” recorded inter-facility transfer arrangements. Unfortunately, details of the planned receiving facilities for transferred patients were not recorded. About 4.51% of this item contained missing data and 8.05% contained a logical mismatch in values between “discharge destination” and “receiving facility”. These records were either corrected or excluded from the final analyses. Hospitals with less than 5 cases of valid recorded “discharge destination” and those with more than 20% of missing values in “discharge destination” were excluded from this study. This resulted in a final sample size of 1,490,695 cases from 604 hospitals. The geographic distribution of the participating hospitals resembles the distribution of all hospitals in the database (Fig. 1).

### Variables and statistical analysis

Inter-facility transfer of patients was the major concern of this study, which was defined as the transfer of a patient from the admitted hospital to another facility for treatment of the same conditions. A code 1 was assigned to a patient who was transferred to another facility at discharge, and 0 otherwise.

Inter-facility transfer rate (IFTR) = transferred out cases recorded in “discharge destination” / all admitted cases with a valid value in “discharge destination” * 100%

We calculated IFTR by population, hospital, and district.

The factors associated with patient transfer were measured at three levels - patients, hospitals, and prefectures. The data structured as patients (first level) nesting within hospitals (second level) and hospitals nesting within prefectures (third level). We performed multilevel logistic regression analyses using MLwiN 2.32 [19]. The first model (Model A) examined the associations between IFTR and all levels of associated variables. We then divided the hospital sample into three subgroups based on their accreditation status and fitted a multilevel model for each group: primary/unrated (Model B1), secondary (Model B2), and tertiary (Model B3).

Independent variables were introduced into the regression models using a stepwise approach. This procedure enabled us to observe changes in variances of random effects at hospital and prefecture levels. Variables examined at the patient level included demographic characteristics of patients (age, sex, and ethnicity), medical insurance, and reasons for hospital admissions (principal diagnosis, comorbidity, admission condition, and surgery). Patients in China were entitled to three medical insurance programs subsidized by the government. Enrollees in the Basic Medical Insurance for Urban Employees (including the retired) enjoyed the highest level of entitlements compared with enrollees of the other two: Basic Insurance for Urban Residents (those who were not employed by the formal public or private sector) and New Rural Cooperative Medical Scheme (for rural residents). Comorbidity was captured in diagnoses other than the principal one. Admission condition was categorized as critical, urgent or non-urgent by the treating medical doctor based on the seriousness of clinical conditions and the urgency for medical interventions at the time of admission. Variables examined at the hospital level included hospital capacity (accredited level) and hospital ownership (public vs. private). Variables examined at the prefecture level included resources (economic zone) and hospital density (number of hospitals per square kilometer).

It is important to adjust for risks of patient transfer. There are several well-established instruments for risk-adjustments: Diagnosis Related Group (DRG) [20], Acute Physiology and Chronic Health Evaluation (APACHE) [21], Case Mix Index (CMI) [22], and Comorbidity Index. The most commonly used instrument is perhaps the DRGs. Unfortunately, a Chinese version of DRGs has not been rigorously contested yet. In this study, we chose the Charlson Comorbidity Index (CCI) [23], which is easy to calculate and has been widely used to reflect the severity and death risk of disease conditions. Up to 17 “other diagnoses” were weighted in the calculation of CCI (Table 3) [24]. The IFTR was adjusted based on the CCI score distribution of all cases in this study.

**Table 3:** Weights assigned to comorbidity conditions

Condition	Weights
Acute myocardial infarction	1
Congestive heart failure	1
Peripheral vascular disease	1
Cerebral vascular accident	1
Dementia	1
Pulmonary disease	1
Connective tissue disorder	1
Peptic ulcer	1
Liver disease	1
Diabetes	1
Diabetes complications	2
Paraplegia	2
Renal disease	2
Cancer	2
Metastatic cancer	6
Severe liver disease	3
HIV	6

All variables were arranged as dichotomous or categorical for the purpose of Chi-square analyses and regression analyses. Patient age was coded into eight groups (0–4, 5–14, 15–29, 30–44, 45–59, 60–69, 70–79, 80+) in line with the categorization adopted in the WHO global burden of disease study [25]. Hospital density was divided into four levels based on quartile and median cut-off points (P25: percentile 25; P50: median; P75: percentile 75) at the county level. CCI was divided into five groups: 0, 1, 2–4, 5–7, 8+ [26].

In the regression analyses, missing data in any of the above variables were denoted as “unknown” and coded as a separate category group. Some groups (such as diagnostic groups or insurance groups) were merged because their IFTRs were similar.

## Results

### Characteristics of hospital patients

More than half (53.15%) of primary/unrated hospitals (*n* = 321) shared only 8.15% (*n* = 121,524) of all admitted cases. By contrast, a small number (*n* = 75, 12.41%) of tertiary hospitals shared 51.71% (*n* = 770,823) of all admitted cases (Table 4).

**Table 4:** Inter-facility transfer of patients by location and context of hospitals

Variable	No. of	No. of	No. of	Transfer rate (%)*	CCI-adjusted rate (%)
	Hospitals (%)	Cases (%)	Transferred (%)		
Economic zone					
One	224 (37.09)	447,567 (30.02)	5039 (16.23)	1.13	1.11
Two	329 (54.47)	946,210 (63.47)	23,607 (76.03)	2.49	2.53
Three	51 (8.44)	96,918 (6.50)	2403 (7.74)	2.48	2.7
Hospital density (number/KM^2^)					
< 0.001	160 (26.49)	66,232 (4.44)	1590 (5.12)	2.4	2.67
[0.001, 0.005)	148 (24.50)	245,273 (16.45)	6112 (19.69)	2.49	2.59
[0.005, 0.013)	152 (25.17)	337,386 (22.63)	10,322 (33.24)	3.06	3.08
≥0.013	144 (23.84)	841,804 (56.47)	13,025 (41.95)	1.55	1.52
Hospital ownership					
Public	279 (46.19)	1,327,274 (89.04)	29,720 (95.72)	2.24	2.24
Private	325 (53.81)	163,421 (10.96)	1329 (4.28)	0.81	0.84
Accredited level of hospitals					
Primary/unrated	321 (53.15)	121,524 (8.15)	1067 (3.44)	0.88	1.3
Secondary	208 (34.44)	598,348 (40.14)	15,544 (50.06)	2.6	3.73
Tertiary	75 (12.42)	770,823 (51.71)	14,438 (46.50)	1.87	1.87

* *P* < 0.001 by Chi-square tests for difference in subgroups of each variable

Gender distribution of the cases was almost equal (49.18% male vs. 50.79% female). The median age of patients was 51 years. Of the admitted patients, 92.70% were Han ethnicity; 71.03% recorded no comorbidity (n = 1,058,825); 5.67% (*n* = 84,529) had a CCI greater than two (see Additional file 1).

### Inter-facility transfer of patients

Inter-facility transfer initiated by doctors only accounted for 2.08% of all admitted cases. In addition, 5.05% cases were discharged against doctors’ advice. No data were recorded about whether those patients sought services from other health facilities.

A total of 195 (32.28%) hospitals recorded zero inter-facility transfer over the study period of the 3 months, 180 of which were primary/unrated; 153 (25.33%) had less than 1% inter-facility transfer; and 10 hospitals had more than 10% inter-facility transfer (the highest was 50.26%).

### Factors associated with inter-facility transfer of patients

#### Univariate analyses

Hospitals located in Economic Zone One had the lowest IFTR. Counties in the P50-P75 hospital density range had the highest IFTR. Public hospitals were more likely to initiate inter-facility patient transfer than their private counterparts. Secondary hospitals recorded the highest IFTR (2.60%); whereas the lowest transfer was found in primary/unrated hospitals. The regional and across-hospital differences in inter-facility transfer of patients became greater after adjusting for CCI (Table 4).

At the patient level, male patients (2.31%) were more likely to be transferred than females (1.86%). The 45–59, 60–69, and 70–79 age groups had a relatively higher IFTR (2.27, 2.42, and 2.38% respectively) while the 15–29 age group had the lowest IFTR (1.60%). The transfer rates of patients who were referred from other facilities (3.04%), and those with critical conditions (4.31%), had no surgery (2.38%), and with a mid-range CCI of 2–4 (3.03%) were higher than the others. Undiagnosed conditions (not elsewhere classified) had the highest IFTR (5.64%) among all diagnosis groups (see Additional file 1).

Patients enrolled with the New Rural Cooperative Medical Scheme (NRCMS) and the Urban Resident Basic Medical Insurance (URBMI) had higher IFTRs (2.37 and 2.15% respectively) than those enrolled with the Urban Employee Basic Medical Insurance (UEBMI) (1.84%) and those with no medical insurance coverage (1.70%). Commercial insurance members recorded an IFTR of 3.91%. A small percentage (1.41%) of patients were covered by “other social insurance” (such as reproductive insurance or work safety insurance). They had the highest IFTR (8.70%), but most were reported from one hospital (see Additional file 1).

#### Multivariate analyses

Model A (Table 5) produced similar results to those of univariate analyses. Most variables identified by the univariate analyses remained statistically significant, except for “ethnicity” and “economic zone”. The odds of inter-facility patient transfer in secondary and tertiary hospitals were 3.55 (*p* < 0.0001) and 2.45 (*p* < 0.0001) times greater than those in primary hospitals, respectively. Such an association was influenced by hospital ownership. Because of the collinearity between hospital ownership and accredited levels of hospitals, hospital ownership was excluded from Model A. Patients without medical insurance coverage (OR = 1.13, *p* < 0.0001) and those covered by URBMI/NRCMS (OR = 1.12, *p* < 0.0001) were more likely to be transferred than those covered by UEBMI. The difference of IFTR between commercial insurance members and UEBMI members was not statistically significant.

**Table 5:** Factors associated with inter-facility patient transfer: results of the multi-level logistic regression analyses

Variables	Model A (All hospitals)	Model B1 (primary/unrated hospitals)	Model B2 (secondary hospitals)	Model B3 (tertiary hospitals)
Fixed effects				
Individual level				
Age (years)(vs. 0–4)				
5–14	1.48 (1.37, 1.60)**	0.77 (0.29, 2.02)	1.13 (1.02, 1.26)	2.10 (1.86, 2.38)**
15–29	2.06 (1.93, 2.19)**	1.44 (0.69, 2.97)	1.49 (1.37, 1.63)**	3.15 (2.85, 3.48)**
30–44	2.22 (2.10, 2.36)**	1.19 (0.58, 2.40)	1.57 (1.45, 1.70)**	3.52 (3.21, 3.85)**
45–59	2.41 (2.28, 2.54)**	1.17 (0.58, 2.33)	1.77 (1.65, 1.90)**	3.69 (3.39, 4.01)**
60–69	2.26 (2.14, 2.40)**	1.12 (0.56, 2.24)	1.64 (1.52, 1.76)**	3.53 (3.24, 3.85)**
70–79	2.04 (1.92, 2.16)**	1.21 (0.60, 2.43)	1.50 (1.39, 1.62)**	3.08 (2.82, 3.37)**
80+	1.63 (1.52, 1.75)**	1.47 (0.72, 2.99)	1.17 (1.07, 1.29)**	2.40 (2.15, 2.67)**
Female (vs. male)	0.85 (0.83, 0.87)**	0.79 (0.68, 0.91)*	0.82 (0.79, 0.86)**	0.88 (0.85, 0.91)**
Minority (vs. Han ethnicity)	1.03 (0.95, 1.12)	0.99 (0.47, 2.07)	0.94 (0.83, 1.07)	1.10 (0.99, 1.22)
Admission type (vs. Emergency department)				
Outpatient clinic	0.82 (0.79, 0.86)**	0.93 (0.74, 1.17)	0.81 (0.77, 0.85)**	0.83 (0.78, 0.88)**
Transferred	3.39 (2.92, 3.93)**	0.71 (0.16, 3.14)	1.36 (1.00, 1.85)*	5.46 (4.63, 6.44)**
Other type or missing	0.94 (0.85, 1.05)	0.37 (0.19, 0.72)*	0.98 (0.85, 1.12)	0.98 (0.84, 1.14)
Condition at admission (vs. non-severe)				
Critical	3.20 (3.03, 3.37)**	4.03 (2.87, 5.68)**	3.26 (3.04, 3.50)**	2.96 (2.74, 3.19)**
Urgent	1.67 (1.61, 1.74)**	1.27 (1.05, 1.54)	1.55 (1.48, 1.64)**	1.82 (1.71, 1.93)**
CCI (score) (vs. 0)				
1	1.18 (1.14, 1.22)**	0.98 (0.80, 1.20)	1.12 (1.07, 1.18)**	1.25 (1.19, 1.32)**
2–4	1.34 (1.29, 1.40)**	1.17 (0.91, 1.50)	1.23 (1.15, 1.30)**	1.45 (1.37, 1.53)**
5–7	1.18 (1.05, 1.32)*	1.72 (0.88, 3.36)	1.52 (1.24, 1.86)**	1.05 (0.91, 1.22)
≥8	1.01 (0.54, 1.88)	1.23 (0.13, 11.2)	2.01 (0.85, 4.70)	0.57 (0.21, 1.56)
Surgical operations (vs. Yes)	1.58 (1.52, 1.64)**	2.38 (1.69, 3.35)**	2.11 (1.97, 2.25)**	1.39 (1.33, 1.46)**
Diagnosis (vs. Not elsewhere classified)				
Infectious/Blood/Endocrine	0.51 (0.47, 0.55)**	0.45 (0.28, 0.73)*	0.43 (0.39, 0.48)**	0.61 (0.55, 0.68)**
Nervous/Perinatal/Circularly/Fatal accident	0.41 (0.38, 0.44)**	0.37 (0.24, 0.56)**	0.40 (0.36, 0.43)**	0.43 (0.39, 0.48)**
Neoplasms/Respiratory/Digestive/Congenital/Injury/services	0.35 (0.32, 0.37)**	0.31 (0.21, 0.47)**	0.31 (0.29, 0.34)**	0.38 (0.35, 0.42)**
Mental/Eye/Ear/Skin/Muscle/Genitourinary/Pregnancy	0.24 (0.22, 0.26)**	0.21 (0.13, 0.32)**	0.23 (0.21, 0.26)**	0.25 (0.23, 0.28)**
Medical insurance (vs. UEBMI)				
URBMI/NRCMS	1.12 (1.07, 1.16)**	0.98 (0.80, 1.20)	1.10 (1.04, 1.17)**	1.17 (1.11, 1.24)**
Commercial insurance	0.91 (0.82, 1.02)	1.41 (0.45, 4.37)	0.95 (0.82, 1.10)	0.85 (0.72, 1.01)
No insurance payment	1.13 (1.07, 1.20)**	1.28 (0.93, 1.75)	1.09 (1.00, 1.19)	1.22 (1.13, 1.31)**
Other social insurance	0.78 (0.72, 0.84)**	0.70 (0.37, 1.33)	1.55 (1.26, 1.90)**	0.75 (0.69, 0.81)**
Other	0.96 (0.90, 1.02)	0.91 (0.66, 1.25)	0.96 (0.87, 1.06)	1.03 (0.95, 1.11)
Hospital level				
Accredited level (vs. primary/unrated)				
Secondary	3.55 (2.63, 4.79)**	–	–	–
Tertiary	2.45 (1.67, 3.59)**	–	–	–
Hospital ownership (vs. public)	–	0.57 (0.27, 1.17)	0.74 (0.48, 1.14)	0.96 (0.05, 15.5)
Region level				
Economic zone (vs. zone one)				
Zone two	0.80 (0.47, 1.35)	0.45 (0.26, 0.77)*	1.27 (0.80, 2.02)	1.20 (0.54, 2.66)
Zone three	1.33 (0.61, 2.92)	0.43 (0.08, 2.28)	2.45 (1.24, 4.87)*	0.49 (0.07, 3.44)
Hospital density (number/km^2^)(vs. < 0.004)				
[0.004, 0.006)	0.69 (0.38, 1.25)	0.38 (0.06, 2.10)	0.70 (0.41, 1.21)	0.27 (0.01, 4.60)
[0.006, 0.009)	0.64 (0.34, 1.18)	0.39 (0.07, 2.07)	0.71 (0.38, 1.30)	0.26 (0.02, 3.27)
≧0.009	0.47 (0.25, 0.89)	0.30 (0.06, 1.55)	0.57 (0.30, 1.09)	0.15 (0.01, 2.26)
Random effect†				
Level 3 region (σ^2^_v0_)‡	0.06 (0.05)	–	–	–
Level 2 hospital (σ^2^_u0_)	1.72 (0.13)**	2.31 (0.33)**	1.20 (0.137)**	1.88 (0.32)**

Notes - Presented by OR (odds ratio) and its 95% CI (confidence intervals); Each diagnosis group was presented with only one word, while complete terms were presented in the supplementary data (see Additional file 1). *UEBMI* Urban Employee Basic Medical Insurance, *URBMI* Urban Resident Basic Medical Insurance, *NRCMS* New Rural Cooperative Medical Scheme

* *P* < 0.01, ** *P* < 0.001

† Estimate and SE

‡ Model A is a three-level model while Models B1–3 are all two-level models without random effect at level 3

Model B2 (for secondary hospitals) and B3 (for tertiary hospitals) revealed similar patient factors predicting IFTRs. However, for primary/unrated hospitals (Model B1), medical insurance and CCI became statistically insignificant in predicting patient transfer. Hospital ownership was no longer a factor predicting patient transfer in Models B1, B2 and B3.

The effects of economic zone on patient transfer varied by level of hospital: for primary/unrated hospitals (Model B1), Zone Two had lower odds (OR = 0.45, *p* = 0.004) than Zone One; for secondary hospitals (Model B2), Zone Three had higher odds (OR = 2.45, *p* = 0.009) than Zone One; for tertiary hospitals (Model B3), there was no significant difference across economic zones.

The random effects of IFTRs across hospitals were large, with an estimated variance of 2.31 for primary/unrated hospitals (*p* < 0.0001), 1.20 for secondary hospitals (*p* < 0.0001) and 1.88 for tertiary hospitals (*p* < 0.0001). This was not entirely explicable by the characteristics of patients and hospitals.

## Discussion

### Main findings

Overall, patient transfer rates are low in general hospitals in Sichuan. On average, 2.08% (31,049) of admitted patients were transferred to other facilities. The CCI-adjusted IFTRs ranged from 1.30% in primary/unrated hospitals, to 1.87% in tertiary hospitals and 3.73% in secondary hospitals. Among these transferred cases, there may be some emergency transfers initiated immediately by the emergency department, which may not necessarily reflect the THCDS arrangements. So the actual transfer rate under the THCDS may be even lower. A national study in the United States revealed that, on average, 4.19% of hospital patients experienced a medical transfer from 2008 to 2011 [27]. Tarima and colleagues found that non-trauma centers in the US transferred 98% of their pediatric patients, even those with very low Injury Severity Score (ISS) [28]. Transfer rates for patients with acute myocardial infarction (AMI) admitted to community hospitals reached 50% in the US [29]. A study using the American National Emergency Department Sample from 2006 to 2012 revealed a 33.71% transfer rate for 1–21 year-old male patients with testicular torsion [30].

The low IFTRs in Sichuan are perhaps associated with the freedom of patient choice of facilities and the lack of trust of patients in primary care facilities. The current healthcare system in China allows patients to choose a tertiary hospital without referral from primary care or a lower level of health provider. Studies show that patients prefer to choose the “right provider” upfront without “being troubled to transfer” at a later stage [31]. Naturally, better-equipped hospitals (often tertiary hospitals) become a preferred option. In a health system where patients trust primary care and use primary care as their first point of contact, patient referral/transfer rates in primary care facilities are usually higher and the lowest patient transfer rate can be found in the higher level health facilities. In the US and UK, for example, inter-hospital patient transfers are a routine and essential part of care in community hospitals [32]. Non-trauma centers, non-teaching hospitals and hospitals in rural areas have higher patient transfer rates than other facilities. Compared with primary care facilities, higher level facilities are more likely to obviate considerations for patient transfer care [13, 33]. Unfortunately, such a system is absent in China.

The low IFTRs could also be an indication of structural problems and a lack of cooperation across health facilities. Health resources have been concentrated in large hospitals in China. China has, on average, 38 hospital beds per 10,000 of the population, compared with 39 in Australia. Despite the high proportion of out-of-pocket payment, China has achieved a 15% hospital admission rate, almost equivalent to Australia’s 16.7% (free in public hospitals) [34, 35]. However, a large proportion (close to half) of these services is provided by tertiary hospitals. Health facilities at lower levels (county and below) are often left under-resourced and under-used. Although the government has recently put great efforts into strengthening primary care, it takes time to get primary care facilities to a status that can attract more patients. For now, hospitals still have strong incentives to compete for patients and increase volumes of services. Tertiary hospitals have greater advantages over their secondary and primary counterparts in the market [8].

This study shows that the severity and complexity of patient conditions could be the major factors associated with the inter-facility patient transfer. Despite differences across different levels of hospitals, higher IFTRs were found consistently for patients who had critical or urgent conditions, or had uncertain diagnoses and patients who were admitted through emergency departments or referred from other facilities. These results are consistent with findings of studies undertaken elsewhere [27, 36]. It is reasonable to predict that it is unlikely that primary and secondary hospitals will refuse to transfer patients whom they are not able to manage properly, and enhancing THCDS may force tertiary hospitals to focus more on the treatment of difficult cases.

Some commentators call for policy incentives for tertiary hospitals to strengthen bonds with secondary and primary hospitals. However, it remains unclear whether the monopoly of tertiary hospitals can be broken through the current policy and insurance interventions [37]. Policies that are put in place for the development of THCDS often include higher insurance reimbursement rates for patients referred from lower levels of health facilities. It is assumed that patients, especially those with limited ability to pay, would take precautions to choose tertiary hospitals as a preferred provider. However, the maximum gap in reimbursement rates among hospitals at different levels is only 7% for UEBMI members and 15% for URBMI/NRCMS members. It is argued that this gap may not be strong enough to encourage patients to divert from tertiary hospitals to secondary or primary hospitals [37].

It seems that higher insurance entitlements discourage inter-facility transfer of inpatients in China. We found that patients without insurance (OR = 1.13) and URBMI/NRCMS members (OR = 1.12) were more likely to experience inter-facility transfer than their UEBMI counterparts. This finding is different from a study in the US using a Nationwide Inpatient Sample (NIS), which found the lowest transfer rate in uninsured patients [36]. Another study in the US using the emergency department database reached the same conclusion [13]. In this study, we found that primary hospitals had the lowest IFTRs. In China, URBMI/NRCMS programs have less funds available and their members enjoy lower levels of entitlements than those enrolled with UEBMI. Patients in China prefer to make tertiary hospitals their first choice of providers. The higher level of insurance entitlements actually enhances the ability of patients to pay for more expensive tertiary hospital services. This helps them realize their first choice, but reduces the possibility of patient transfer from primary hospitals to secondary/tertiary hospitals. Previous studies in China showed consumers are not aware of the policies in relation to THCDS and there is a lack of trust in primary care providers. This further exaggerates patient preference for tertiary hospitals [31].

### Strengths

To our knowledge, this is the first study of its kind in China to examine inter-facility patient transfer using a large sample of inpatient records. We performed multilevel modelling, analyzing variables that are associated with patient transfer at three levels: patient characteristics (age, gender, insurance, admission pattern, diagnoses, medical procedures), hospital characteristics (ownership and accredited level), and location of hospitals (economic and resources). The study has policy implications for the development of THCDS.

### Limitations and further study

This study had several limitations. Firstly, the study adopted a cross-sectional design, hence it is not able to capture changing volumes of patients across different levels of hospitals. Secondly, the patient transfer data did not point to any directions. We do not know whether a patient was transferred to a higher level of hospital or a lower level of hospital. In addition, the study did not tap into non-admitted patient care. Thirdly, some hospitals had not implemented an electronic medical record system and were excluded from this study. As a result, the IFTRs could be biased or not entirely representative of the province. Fourthly, the CCI adjustment method considered a limited number of comorbidity. Future studies should explore more complicated methods of risk adjustments. Finally, patient care outcomes were not included in data analyses due to difficulties to link patient identity with medical records across hospitals.

## Conclusion

International experience shows that a primary-care-dominated THCDS is the most cost efficient and cost effective service. The health system reform in China is heading in the right direction but is facing great challenges. The current system is still dominated by tertiary hospitals. There is a shortage of inter-facility cooperation and patient transfer. The development of social health insurance, which covers over 96% of the population, and increased insurance benefits have failed to encourage patients to choose primary and secondary facilities as their preferred providers. There is a concern that the different social insurance programs may widen the inequity in hospital care. As shown in this study, patients enrolled in the most generous insurance scheme, UEBMI, are more likely to be admitted to tertiary hospitals and least likely to transfer to other facilities compared with their URBMI/NRCMS counterparts and those without insurance.

Strengthening the capacity of primary and secondary facilities is critical in the development of THCDs. However, it is equally important, if not more, to align insurance policies with the goals of THCDs. The capacity control of tertiary hospital services should be considered, either through stronger planning or through reforms of funding mechanisms (such as a casemix payment system) or both. Patient support is also essential.

## Additional file

Additional file 1: **Table S1.** Patient characteristics associated with inter-facility patient transfer. (DOCX 26 kb)

## Abbreviations

95% CI: 95% confidence interval
AMI: Acute myocardial infarction
APACHE: Acute Physiology and Chronic Health Evaluation
UEBMI: Urban Employee Basic Medical Insurance
URBMI: Urban Resident Basic Medical Insurance
CCI: Charlson Comorbidity Index
CMI: Case Mix Index
DRG: Diagnosis Related Group
IFTR: Inter-facility transfer rate
ISS: Injury Severity Score
NRCMS: New rural cooperative medical scheme
OR: Odds ratio
P25: Percentile 25
P50: Median
P75: Percentile 75
THCDS: Tiered health care delivery system
