# Supplementary material for: Factors associated with the inter-facility transfer of inpatients in Sichuan province, China
Source: BMC Health Serv Res. 2019 May 23;19:329. doi: 10.1186/s12913-019-4153-7 (PMC6533730; doi:10.1186/s12913-019-4153-7)
Supplement: Supplementary file 1 — Table S1. Patient characteristics associated with inter-facility patient transfer. (DOCX 26 kb) [file 12913_2019_4153_MOESM1_ESM.docx]

Supplementary data

Attached table 1. Patient characteristics associated with inter-facility patient transfer

| **Characteristics** | **All hospitals** | |  | **Primary/unrated hospital** | |  | **Secondary hospital** | |  | **Tertiary hospital** | |
| --- | --- | --- | --- | --- | --- | --- | --- | --- | --- | --- | --- |
|  | **No. of cases** | **No. of   Transferred (%)** |  | **No. of cases** | **No. of Transferred (%)** |  | **No. of cases** | **No. of Transferred (%)** |  | **No. of cases** | **No. of Transferred (%)** |
| **Sex** |  |  |  |  |  |  |  |  |  |  |  |
| Male | 733075 | 16955(2.31) |  | 54883 | 542(0.99) |  | 292941 | 8524(2.91) |  | 385251 | 7889(2.05) |
| Female | 757063 | 14057(1.86) |  | 66479 | 521(0.78) |  | 305103 | 6987(2.29) |  | 385481 | 6549(1.7) |
| **Age** |  |  |  |  |  |  |  |  |  |  |  |
| 0-4 | 153832 | 2483(1.61) |  | 2195 | 12(0.55) |  | 72306 | 1565(2.16) |  | 79331 | 906(1.14) |
| 5-14 | 56116 | 1116(1.99) |  | 2137 | 10(0.47) |  | 26905 | 619(2.3) |  | 27074 | 487(1.8) |
| 15-29 | 168093 | 2693(1.6) |  | 10282 | 64(0.62) |  | 74106 | 1458(1.97) |  | 83705 | 1171(1.4) |
| 30-44 | 215045 | 4033(1.88) |  | 19031 | 120(0.63) |  | 83246 | 1844(2.22) |  | 112768 | 2069(1.83) |
| 45-59 | 311329 | 7067(2.27) |  | 30227 | 231(0.76) |  | 112024 | 3247(2.9) |  | 169078 | 3589(2.12) |
| 60-69 | 265596 | 6424(2.42) |  | 27848 | 258(0.93) |  | 99744 | 3070(3.08) |  | 138004 | 3096(2.24) |
| 70-79 | 217398 | 5181(2.38) |  | 20644 | 227(1.1) |  | 88118 | 2692(3.05) |  | 108636 | 2262(2.08) |
| 80+ | 102834 | 2050(1.99) |  | 9154 | 145(1.58) |  | 41720 | 1048(2.51) |  | 51960 | 857(1.65) |
| **Ethnicity** |  |  |  |  |  |  |  |  |  |  |  |
| Han | 1381936 | 28941(2.09) |  | 114670 | 1016(0.89) |  | 542668 | 14477(2.67) |  | 724598 | 13448(1.86) |
| Minority | 70046 | 1465(2.09) |  | 4904 | 35(0.71) |  | 37767 | 796(2.11) |  | 27375 | 634(2.32) |
| **Medical insurance** |  |  |  |  |  |  |  |  |  |  |  |
| UEBMI | 332717 | 6134(1.84) |  | 31005 | 318(1.03) |  | 106991 | 2117(1.98) |  | 194721 | 3699(1.9) |
| URBMI | 206533 | 4442(2.15) |  | 31742 | 294(0.93) |  | 76075 | 1602(2.11) |  | 98716 | 2546(2.58) |
| NRCMS | 498080 | 11813(2.37) |  | 34456 | 188(0.55) |  | 261718 | 8584(3.28) |  | 201906 | 3041(1.51) |
| Medical assistance | 5334 | 87(1.63) |  | 1015 | 1(0.1) |  | 2608 | 39(1.5) |  | 1711 | 47(2.75) |
| Commercial insurance | 19334 | 755(3.91) |  | 297 | 4(1.35) |  | 8062 | 522(6.47) |  | 10975 | 229(2.09) |
| Free medicine | 31768 | 456(1.44) |  | 2509 | 24(0.96) |  | 8350 | 118(1.41) |  | 20909 | 314(1.5) |
| No insurance payment | 196051 | 3329(1.7) |  | 7676 | 130(1.69) |  | 69716 | 1459(2.09) |  | 118659 | 1740(1.47) |
| Other social insurance | 21035 | 1829(8.7) |  | 3299 | 25(0.76) |  | 4334 | 123(2.84) |  | 13402 | 1681(12.54) |
| Else | 179843 | 2204(1.23) |  | 9525 | 83(0.87) |  | 60494 | 980(1.62) |  | 109824 | 1141(1.04) |
| **Admitted through** |  |  |  |  |  |  |  |  |  |  |  |
| Emergency department | 361858 | 7756(2.14) |  | 17692 | 232(1.31) |  | 152941 | 5005(3.27) |  | 191225 | 2519(1.32) |
| Outpatient clinic | 828931 | 13770(1.66) |  | 88480 | 781(0.88) |  | 340221 | 9047(2.66) |  | 400230 | 3942(0.98) |
| Transferred | 8496 | 258(3.04) |  | 2757 | 4(0.15) |  | 2924 | 57(1.95) |  | 2815 | 197(7) |
| Others | 291410 | 9265(3.18) |  | 12595 | 50(0.4) |  | 102262 | 1435(1.4) |  | 176553 | 7780(4.41) |
| **Diagnosis** |  |  |  |  |  |  |  |  |  |  |  |
| I Certain infectious and parasitic diseases | 49470 | 1498(3.03) |  | 1384 | 10(0.72) |  | 18931 | 689(3.64) |  | 29155 | 799(2.74) |
| II Neoplasms | 85198 | 1768(2.08) |  | 2617 | 32(1.22) |  | 21184 | 678(3.2) |  | 61397 | 1058(1.72) |
| III Diseases of the blood and blood-forming organs and certain disorders involving the immune mechanism | 11731 | 409(3.49) |  | 215 | 5(2.33) |  | 3513 | 199(5.66) |  | 8003 | 205(2.56) |
| IV Endocrine, nutritional and metabolic diseases | 35575 | 1158(3.26) |  | 1611 | 55(3.41) |  | 11376 | 402(3.53) |  | 22588 | 701(3.1) |
| V Mental and behavioral disorders | 11645 | 186(1.6) |  | 1478 | 2(0.14) |  | 3440 | 82(2.38) |  | 6727 | 102(1.52) |
| VI Diseases of the nervous system | 38850 | 1048(2.7) |  | 2832 | 25(0.88) |  | 13719 | 336(2.45) |  | 22299 | 687(3.08) |
| VII Diseases of the eye and adnexa | 36653 | 304(0.83) |  | 1964 | 2(0.1) |  | 12041 | 130(1.08) |  | 22648 | 172(0.76) |
| VIII Diseases of the ear and mastoid process | 14165 | 237(1.67) |  | 1128 | 6(0.53) |  | 5864 | 129(2.2) |  | 7173 | 102(1.42) |
| IX Diseases of the circulatory system | 181117 | 5114(2.82) |  | 16913 | 210(1.24) |  | 73849 | 2943(3.99) |  | 90355 | 1961(2.17) |
| X Diseases of the respiratory system | 279852 | 5037(1.8) |  | 28218 | 240(0.85) |  | 129898 | 2793(2.15) |  | 121736 | 2004(1.65) |
| XI Diseases of the digestive system | 198666 | 4414(2.22) |  | 16508 | 189(1.14) |  | 82936 | 2313(2.79) |  | 99222 | 1912(1.93) |
| XII Diseases of the skin and subcutaneous tissue | 16695 | 259(1.55) |  | 1363 | 9(0.66) |  | 5520 | 147(2.66) |  | 9812 | 103(1.05) |
| XIII Diseases of the musculoskeletal system and connective tissue | 78330 | 1004(1.28) |  | 17794 | 86(0.48) |  | 28847 | 484(1.68) |  | 31689 | 434(1.37) |
| XIV Diseases of the genitourinary system | 111434 | 1544(1.39) |  | 15927 | 84(0.53) |  | 41878 | 695(1.66) |  | 53629 | 765(1.43) |
| XV Pregnancy, childbirth and the puerperium | 89751 | 900(1) |  | 2648 | 15(0.57) |  | 45445 | 622(1.37) |  | 41658 | 263(0.63) |
| XVI Certain conditions originating in the perinatal period | 19626 | 511(2.6) |  | 23 | 1(4.35) |  | 7632 | 312(4.09) |  | 11971 | 198(1.65) |
| XVII Congenital malformations, deformations and chromosomal abnormalities | 5813 | 112(1.93) |  | 105 | 1(0.95) |  | 1009 | 27(2.68) |  | 4699 | 84(1.79) |
| XVIII Symptoms, signs and abnormal clinical and laboratory findings, not elsewhere classified | 25046 | 1412(5.64) |  | 1046 | 46(4.4) |  | 10189 | 784(7.69) |  | 13811 | 582(4.21) |
| XIX Injury, poisoning and certain other consequences of external causes | 126954 | 2783(2.19) |  | 4831 | 28(0.58) |  | 66092 | 1580(2.39) |  | 56031 | 1175(2.1) |
| XX External causes of morbidity and mortality | 946 | 24(2.54) |  | 370 | 6(1.62) |  | 495 | 17(3.43) |  | 81 | 1(1.23) |
| XXI Factors influencing health status and contact with health services | 66712 | 1262(1.89) |  | 1370 | 11(0.8) |  | 14325 | 181(1.26) |  | 51017 | 1070(2.1) |
| **Condition at admission** |  |  |  |  |  |  |  |  |  |  |  |
| Critical | 61125 | 2634(4.31) |  | 2127 | 74(3.48) |  | 21201 | 1521(7.17) |  | 37797 | 1039(2.75) |
| Urgent | 244276 | 5870(2.4) |  | 23885 | 261(1.09) |  | 99089 | 3585(3.62) |  | 121302 | 2024(1.67) |
| Not severe | 916231 | 16127(1.76) |  | 92454 | 705(0.76) |  | 332249 | 6973(2.1) |  | 491528 | 8449(1.72) |
| **Critical conditions after admission** |  |  |  |  |  |  |  |  |  |  |  |
| Yes | 205077 | 8504(4.15) |  | 11193 | 222(1.98) |  | 70070 | 4709(6.72) |  | 123814 | 3573(2.89) |
| No | 1285618 | 22545(1.75) |  | 110331 | 845(0.77) |  | 528278 | 10835(2.05) |  | 647009 | 10865(1.68) |
| **Department** |  |  |  |  |  |  |  |  |  |  |  |
| Internal Medicine | 521723 | 13838(2.65) |  | 55929 | 651(1.16) |  | 208379 | 7497(3.6) |  | 257415 | 5690(2.21) |
| Surgery | 355287 | 6214(1.75) |  | 22717 | 137(0.6) |  | 145219 | 3224(2.22) |  | 187351 | 2853(1.52) |
| Obstetrics and Gynecology | 128442 | 1224(0.95) |  | 10202 | 38(0.37) |  | 57431 | 785(1.37) |  | 60809 | 401(0.66) |
| Pediatrics | 134026 | 2218(1.65) |  | 607 | 5(0.82) |  | 61177 | 1425(2.33) |  | 72242 | 788(1.09) |
| Emergency Medicine | 3848 | 297(7.72) |  | . | . |  | 2940 | 255(8.67) |  | 908 | 42(4.63) |
| Critical Care | 5572 | 429(7.7) |  | 56 | 7(12.5) |  | 1492 | 225(15.08) |  | 4024 | 197(4.9) |
| Infectious Diseases | 19807 | 589(2.97) |  | 17 | 0(0) |  | 7297 | 204(2.8) |  | 12493 | 385(3.08) |
| Oncology | 33159 | 971(2.93) |  | 453 | 3(0.66) |  | 4120 | 20(0.49) |  | 28586 | 948(3.32) |
| Others | 288831 | 5269(1.82) |  | 31543 | 226(0.72) |  | 110293 | 1909(1.73) |  | 146995 | 3134(2.13) |
| **Surgical operations** |  |  |  |  |  |  |  |  |  |  |  |
| Yes | 535852 | 8319(1.55) |  | 17618 | 51(0.29) |  | 226430 | 4460(1.97) |  | 291804 | 3808(1.3) |
| No/missing | 954843 | 22730(2.38) |  | 103906 | 1016(0.98) |  | 371918 | 11084(2.98) |  | 479019 | 10630(2.22) |
| **CCI** |  |  |  |  |  |  |  |  |  |  |  |
| 0 | 1058825 | 19032(1.8) |  | 88436 | 745(0.84) |  | 444301 | 10166(2.29) |  | 526088 | 8121(1.54) |
| 1 | 239924 | 6373(2.66) |  | 22072 | 181(0.82) |  | 94071 | 3294(3.5) |  | 123781 | 2898(2.34) |
| 2-4 | 173847 | 5259(3.03) |  | 10434 | 128(1.23) |  | 55871 | 1951(3.49) |  | 107542 | 3180(2.96) |
| 5-7 | 17382 | 373(2.15) |  | 544 | 12(2.21) |  | 3888 | 126(3.24) |  | 12950 | 235(1.81) |
| ≥8 | 717 | 12(1.67) |  | 38 | 1(2.63) |  | 217 | 7(3.23) |  | 462 | 4(0.87) |

**Note: UEBMI: U**rban Employee Basic Medical Insurance; **URBMI: U**rban Resident Basic Medical Insurance; **NRCMS: N**ew Rural Cooperative Medical Scheme.

**Diagnosis is based on the International Statistical Classification of Diseases and Related Health Problems 10th Revision (ICD-10)-WHO Version.**
